# The prognostic value of plasma fibrinogen levels in patients with endometrial cancer: a multi-centre trial

**DOI:** 10.1038/sj.bjc.6605547

**Published:** 2010-02-16

**Authors:** V Seebacher, S Polterauer, C Grimm, H Husslein, H Leipold, K Hefler-Frischmuth, C Tempfer, A Reinthaller, L Hefler

**Affiliations:** 1 Department of Obstetrics and Gynaecology, Medical University of Vienna, Waehringer Guertel 18-20, Vienna 1090, Austria; 2 Department of Obstetrics and Gynaecology, Landeskrankenhaus Klagenfurt, Krassnigstraße, 9020 Klagenfurt, Carinthia, Austria; 3 Department of Laboratory Medicine, Wilhelminenspital, Montleartstrasse 37, 1160, Vienna, Austria

**Keywords:** fibrinogen, endometrial cancer, prognosis

## Abstract

**Background:**

To analyse the correlation between pre-treatment plasma fibrinogen levels and clinical–pathological parameters in patients with endometrial cancer and to assess the value of plasma fibrinogen as a prognostic parameter.

**Methods:**

Within a retrospective multi-centre study, the records of 436 patients with endometrial cancer were reviewed and pre-treatment plasma fibrinogen levels were correlated with clinical–pathological parameters and patients’ survival.

**Results:**

The mean (s.d.) pre-treatment plasma fibrinogen level was 388.9 (102.4) mg per 100 ml. Higher plasma fibrinogen levels were associated with advanced tumour stage (FIGO I *vs* II *vs* III and IV, *P*=*0.002*), unfavourable histological subtype (endometrioid *vs* non-endometrioid histology, *P*=0.03), and higher patients’ age (⩽67 years *vs* >67 years, *P*=*0.04*), but not with higher histological grade (G1 *vs* G2 *vs* G3, *P*=*0.2*). In a multivariate analysis, tumour stage (*P*<*0.001* and *P*<*0.001*), histological grade (*P*=*0.009* and *P*=*0.002*), patients’ age (*P*=*0.001* and *P*<*0.001*), and pre-treatment plasma fibrinogen levels (*P*=*0.04* and *P*=*0.02*) were associated with disease-free and overall survival, respectively.

**Conclusion:**

Plasma fibrinogen levels can be used as an independent prognostic parameter for the disease-free and overall survival of patients with endometrial cancer.

The association between cancer and haemostasis, as well as inflammation, is widely accepted (Dvorak, 1986; Andreasen *et al*, 2000; Balkwill and Mantovani, 2001; Coussens and Werb, 2002). A number of procoagulant and fibrinolytic factors have been found to be overexpressed in malignant human and animal tumour cells (Dvorak, 1986; Brown *et al*, 1988; Bardos *et al*, 1996).

One of these factors is fibrinogen, a plasma glycoprotein that is mainly responsible for the formation of a meshwork of fibrin monomers that consolidates an initial platelet plug into a solid haemostatic clot (Hantgan *et al*, 2001). As one of several acute-phase reactant proteins, it rises during systemic inflammation and tissue injury (Holm and Godal, 1984). Fibrinogen is mainly produced by hepatocytes; however, extrahepatical synthesis by epithelial cells and tumour cells has been described (Lawrence and Simpson-Haidaris, 2004).

Extensive studies on human and animal tumour biology indicate a specific link between fibrinogen and the progressive and metastatic behaviour of tumour cells (Palumbo *et al*, 2000, 2002, 2005; Simpson-Haidaris and Rybarczyk, 2001). Recent studies showed a correlation between elevated plasma fibrinogen levels and tumour progression in patients with gastric (Lee *et al*, 2004; Yamashita *et al*, 2005, 2006), oesophageal (Takeuchi *et al*, 2007), non-small-cell lung (Jones *et al*, 2006), breast, ovarian (Von Tempelhoff *et al*, 2000), and cervical cancer (Polterauer *et al*, 2009).

Regarding endometrial cancer, a hypercoagulable state is known to be associated with advanced tumour stage and higher histological grade (Von Tempelhoff *et al*, 2000). In a small series of patients with endometrial cancer (*n*=70), pre-treatment plasma fibrinogen levels correlated with tumour stage but not with patients’ survival (Von Tempelhoff *et al*, 2000). The aim of this study was to analyse the correlation between pre-treatment plasma fibrinogen levels and clinical–pathological parameters in patients with endometrial cancer and to assess the value of plasma fibrinogen as a prognostic parameter in a large multi-centre trial.

## Materials and methods

### Patients

A total of 436 consecutive patients with endometrial cancer, treated at the Departments of Obstetrics and Gynaecology at the Medical University of Vienna (*n*=360) and at the Landeskrankenhaus Klagenfurt (*n*=76) between December 1995 and June 2008, were enrolled in this study. Clinical and laboratory data were extracted retrospectively from patient files.

### Clinical management

Diagnosis of endometrial cancer was established by dilation and curettage. Subsequently, patients were surgically staged according to the International Federation of Gynaecology and Obstetrics (FIGO)/American Joint Committee on Cancer (AJCC) classification system.

Hysterectomy, bilateral salpingo-oophorectomy, cytological examination of peritoneal fluid, and biopsy of any suspicious intraperitoneal or retroperitoneal lesions were performed. Pelvic and paraaortic lymphadenectomy was conducted, except for tumour stage Ia and Ib with histological grades 1 and 2 and endometrioid histology. In patients with intermediate-risk or high-risk disease, adjuvant radiotherapy was provided according to standardised treatment protocols (Nag *et al*, 2000). A regimen of adjuvant chemotherapy using carboplatin/paclitaxel was used in selected patients with advanced disease.

At 3 months after completion of primary therapy, the first follow-up visit was scheduled. Patients had follow-up visits at 3-month intervals for the following 3 years, including inspection, vaginal–rectal palpation, and serum tumour marker evaluation. In the fourth and fifth year, visits were scheduled bi-annually, and once a year from the sixth to the tenth year after primary therapy. When patients did not present for scheduled follow-up visits, they were contacted by administrative personnel or nurses. When any clinically suspicious finding and/or tumour marker elevation was observed, computed tomography was performed. Recurrent disease was diagnosed histologically or by the presence of a measurable lesion on computed tomography.

### Measurement of fibrinogen and other laboratory parameters

The analysis of plasma fibrinogen levels, serum CA 125, white blood cell count, and platelet count was routinely performed before therapy. Blood samples (citrated plasma) were obtained by peripheral venous puncture before surgery. Plasma fibrinogen levels were determined by the Clauss method (Clauss, 1957) using clotting reagents from Diagnostica Stago (Asnieres, France). The manufacturer claims an intra-assay variability of 3.5%. Plasma fibrinogen levels between 180 and 390 mg per 100 ml were defined as normal. For serum CA 125, a cutoff value of 40 U ml^–1^ has been determined, as described previously (Hsieh *et al*, 2002). Cutoff values for white blood cell count (1 × 10^10^ *μ*l^–1^) and platelet count (450 000 *μ*l^–1^) were used according to standardised guidelines for leukocytosis and thrombocytosis.

### Statistical analysis

Values are given as means (s.d.). The *t*-tests and one-way ANOVA were used to compare fibrinogen plasma levels and clinical–pathological parameters. Survival probabilities were calculated by the product limit method of Kaplan–Meier. Differences between groups were tested using the log-rank test. Results were analysed for the end point of disease-free and overall survival. Events were defined as death or progression at the time of the last follow-up visit. Survival times of disease-free patients or patients with stable disease were censored with the last follow-up-date, and patients with death causes other than endometrial cancer were censored with the date of death. Univariate and multivariate Cox regression models for disease-free and overall survival were performed, comprising FIGO tumour stage (FIGO I *vs* FIGO II *vs* FIGO III and IV), histological grade (G1 *vs* G2 *vs* G3), histological subtype (endometrioid adenocarcinoma *vs* non-endometrioid carcinoma, such as serous and clear cell carcinoma), and pre-treatment plasma fibrinogen levels. The *P*-values of <0.05 were considered statistically significant. We used the statistical software SPSS 16.0 for Mac (SPSS 16.0.1, SPSS Inc., Chicago, IL, USA) for statistical analysis.

### Institutional review board

This study was approved by our institutional review board, by the Ethics-Committee of the Medical University of Vienna, and by the Vienna General Hospital (AKH).

## Results

Patients’ characteristics are given in Table 1. The mean (s.d.) pre-treatment plasma fibrinogen level was 388.9 (102.4) mg per 100 ml. Lymph node status was available in 180 patients. Lymph node involvement was noted in 26 patients. In all, 168 patients received adjuvant radiotherapy, and 35 patients received adjuvant chemotherapy.

The correlation between pre-treatment plasma fibrinogen levels and clinical–pathological parameters is given in Table 2. Higher pre-treatment plasma fibrinogen levels were associated with advanced tumour stage, unfavourable histological subtype, and higher patients’ age, but not with higher histological grade. Pre-treatment plasma fibrinogen levels were significantly associated with serum CA 125, white blood cell count, and platelet count (Table 2).

**Figure 1:**
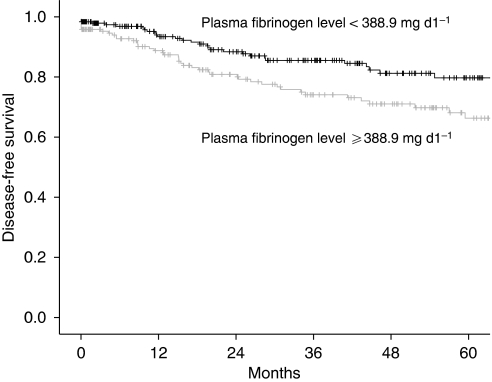
Kaplan–Meier curves for disease-free survival broken down by the mean plasma fibrinogen level.

**Figure 2:**
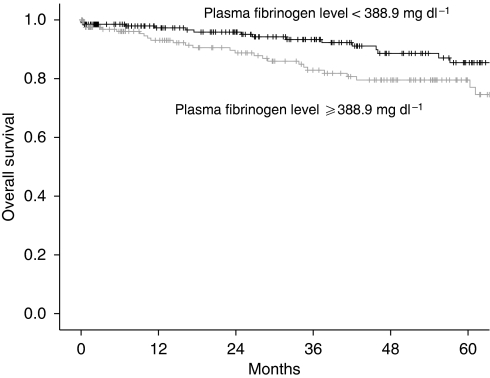
Kaplan–Meier curves for overall survival broken down by the mean plasma fibrinogen level.

In a univariate analysis, tumour stage, histological grade, histological subtype, patients’ age, and pre-treatment plasma fibrinogen levels were associated with disease-free and overall survival. In a multivariate analysis, tumour stage, histological grade, patients’ age, and pre-treatment plasma fibrinogen levels were associated with disease-free and overall survival. Results of the univariate and multivariate Cox regression models and log-rank tests with respect to overall and disease-free survival are shown in Table 3. It is noteworthy that when patients were grouped according to the mean plasma fibrinogen level, patients with plasma fibrinogen levels of <388.9 and ⩾388.9 mg per 100 ml had a 5-year disease-free survival rate of 80 and 66% (P=0.01) and an overall survival rate of 84 and 74% (P=0.005), respectively (Figures 1 and 2). By comparing pre-treatment plasma fibrinogen levels between patients with and without recurrent disease in each FIGO tumour stage separately, we could show a significant difference within the group of FIGO tumour stage III and IV (*P*=0.03), but not within the group of FIGO tumour stage I (*P*=0.3) and II (*P*=0.5).

## Discussion

A strong association between haemostasis and tumour biology has been described previously (Dvorak, 1986; Von Tempelhoff *et al*, 2000; Balkwill and Mantovani, 2001; Coussens and Werb, 2002). In this retrospective multi-centre trial, we determined the effect of pre-treatment plasma fibrinogen levels on endometrial cancer in a large series of patients. By correlating pre-treatment plasma fibrinogen levels with clinical–pathological parameters, we could show a significant association between elevated plasma fibrinogen levels and advanced tumour stage.

These findings might be explained by hyperfibrinogenaemia induced by an inflammatory reaction to tumour growth and a hypercoagulable state in cancer patients (Gouin-Thibault *et al*, 2001; Coussens and Werb, 2002). Other studies describe the endogenous production of fibrinogen by tumour cells themselves (Lee *et al*, 1996; Lawrence and Simpson-Haidaris, 2004; Sahni *et al*, 2008). Fibrinogen and its degradation product fibrin seem to encase tumour cells and are capable of binding numerous cell types and growth factors, and exert an effect as a bridge between tumour and epithelial cells, thus providing structure to the tumour stroma and altering invasive potential (Dvorak *et al*, 1983; Dvorak, 1986; Costantini *et al*, 1991; Simpson-Haidaris and Rybarczyk, 2001). Through an interaction with fibroblast growth factor-2 (FGF-2) and vascular endothelial growth factor (VEGF), fibrinogen promotes angiogenesis and tumour cell growth (Sahni and Francis, 2000; Sahni *et al*, 2006, 2008). Another interesting research question would be to compare plasma fibrinogen levels before surgery, after surgery, during follow-up, and in an eventual recurrent disease situation. These questions cannot be answered by our present study, as only pre-treatment plasma fibrinogen levels were determined.

Not only tumour growth but also the metastatic potential of tumour cells seems to be influenced by fibrinogen, as could be showed in mice model studies (Palumbo *et al*, 2000, 2002, 2005). In our study, we could show a significant correlation between pre-treatment plasma fibrinogen levels and pre-treatment CA 125 levels. As CA 125 has been associated with lymphatic tumour cell spread in patients with endometrial cancer (Hsieh *et al*, 2002), our results seem to underline a possible functional link between fibrinogen and metastasis. Fibrinogen seems to stabilise tumour-associated platelets building microthrombi, which exert an effect as a physical barrier, preventing adherence of natural killer cells, monocytes, and lymphocytes to tumour cells. By protecting tumour cells against the innate immune system, fibrinogen enhances their metastatic potential (Palumbo *et al*, 2000, 2002, 2005; Biggerstaff *et al*, 2006, 2008).

The most interesting result of our study was the prognostic value of pre-treatment plasma fibrinogen levels regarding patients’ survival. The prognosis of patients with endometrial cancer is primarily determined by tumour stage, histological grade, histological subtype, and patients’ age (Creasman *et al*, 2001; Jolly *et al*, 2006; Fujimoto *et al*, 2009). Only limited data exist on prognostic serum tumour markers in patients with endometrial cancer, such as CA 125 and C-reactive protein (Dotters, 2000; Schmid *et al*, 2007). An association between blood rheology and prognosis in patients with gynaecological malignancy has previously been reported (Von Tempelhoff *et al*, 2000). Fibrinogen is associated with high plasma viscosity; it promotes aggregation of red blood cells and decreases cell deformability, resulting in reduced blood flow and oxygen transport capacity (Lowe, 1994). Low oxygenation of tumour tissue has been shown to be indicative of resistance to radiotherapy and poor prognosis on overall survival in cervical cancer (Hockel *et al*, 1996; Gundersen and Palmer, 2008).

One of the reviewers of this paper pointed us towards another unusual but interesting analysis. We compared pre-treatment plasma fibrinogen levels between non-recurrent and recurrent groups of patients in each FIGO tumour stage. A significant difference was only shown for patients with advanced stage, but not for early-stage disease. These findings are interesting, but do not change the conclusions of our study, as the multivariate Cox regression analysis confirmed the independence of the association between plasma fibrinogen levels and patients’ survival. Presumably in early FIGO tumour stages, the number of patients with recurrent disease is too small for showing a statistically significant difference.

Owing to early diagnosis of endometrial cancer, the overall prognosis of patients is relatively good compared with other gynaecological malignancies. Nevertheless, it is important to identify prognostic parameters in selecting patients for adjuvant therapy to avoid over-treatment. Along with pre-treatment plasma fibrinogen levels, the established prognostic parameters, namely, tumour stage, histological grade, and patients’ age, were found to be independently associated with survival. The majority of recurrence occurs within 36 months after diagnosis, which is covered by the follow-up time of the patients involved in our study (Fung-Kee-Fung *et al*, 2006).

In summary, our results show that plasma fibrinogen level is an independent prognostic parameter in patients with endometrial cancer. By showing a correlation between higher pre-treatment plasma fibrinogen levels and advanced tumour stage, we provide some insight into the possible functional properties of fibrinogen.

## Figures and Tables

**Table 1:** Patients’ characteristics

**Parameter**	**Number (%) or mean (s.d.)**
Total number of patients enrolled	436
Age at first diagnosis (years)	66.9 (11.2)
	
*Tumour stage*	
FIGO Ia	69 (15.8)
FIGO Ib	191 (43.8)
FIGO Ic	67 (15.4)
FIGO IIa	17 (3.9)
FIGO IIb	23 (5.3)
FIGO IIIa	29 (6.7)
FIGO IIIb	4 (0.9)
FIGO IIIc	24 (5.5)
FIGO IVa	3 (0.7)
FIGO IVb	9 (2.0)
	
*Histological grade*	
G1	190 (43.6)
G2	151 (34.6)
G3	95 (21.8)
	
*Histological subtype*	
Endometrioid histology	408 (93.6)
Non-endometrioid histology (clear cell, serous carcinoma)	28 (6.4)
Time of follow-up (months)	36.1 (31.1)
	
*Recurrence status*	
Number of patients with recurrent disease	76 (17.4)
Time to recurrent disease (months)	19.9 (18.9)
	
*Status at last observation*	
Disease-free	340 (78.0)
Stable disease	22 (5.0)
Progressive disease	42 (9.6)
Tumour-related death	20 (4.6)
Death related to other causes	12 (2.8)

Abbreviation: FIGO=International Federation of Gynecology and Obstetrics.

**Table 2:** Mean plasma fibrinogen levels in patients with endometrial cancer broken down by clinical–pathological and laboratory parameters

	**Mean (s.d.) plasma fibrinogen levels (mg per 100** **ml)**	***P*-value**
*FIGO tumour stage* a		0.002
I	379.7 (99.6)	
II	400.4 (87.8)	
III and IV	425.8 (114.9)	
		
*Histological grade* a		0.2
G1	385.8 (104.8)	
G2	383.2 (99.3)	
G3	405.6 (100.9)	
		
*Histological subtype* b		0.03
Endometrioid histology	386.1 (98.5)	
Non-endometrioid histology	429.1 (144.8)	
		
*Age* b		0.04
⩽67 years	379.3 (100.3)	
>67 years	398.9 (103.8)	
		
*CA 125* b		0.02
⩽40 U ml^–1^	399.6 (87.7)	
>40 U ml^–1^	432.2 (124.8)	
		
*White blood cells* b		0.002
⩽1 × 10^10^ *μ*l^–1^	384.2 (97.4)	
>1 × 10^10^ *μ*l^–1^	442.7 (129.6)	
		
*Platelets* b		<0.001
⩽450 000 *μ*l^–1^	385.3 (96.9)	
>450 000 *μ*l^–1^	563.0 (163.04)	

Abbreviation: FIGO=International Federation of Gynecology and Obstetrics.

a One-way ANOVA.

b Independent two-sided *t*-test.

**Table 3:** Univariate and multivariate survival analysis

	**Disease-free survival**				**Overall survival**			
	**Univariate analysis^a^**		**Multivariate analysis^b^**		**Univariate analysis^a^**		**Multivariate analysis^b^**	
	***P*-value**	**HR (95% CI)**	***P*-value**	**HR (95% CI)**	***P*-value**	**HR (95% CI)**	***P*-value**	**HR (95% CI)**
Tumour stage^c^	<0.001	—	<0.001	2.2 (1.7–2.8)	<0.001	—	<0.001	2.1 (1.6–2.8)
Histological grade^d^	<0.001	—	0.04	1.4 (1.02–2.0)	<0.001	—	0.002	1.8 (1.2–2.5)
Histological subtype^e^	<0.001	—	0.1	1.7 (0.9–3.4)	0.002	—	0.6	1.3 (0.6–2.8)
Age	<0.001	1.1 (1.0–1.1)	0.001	1.04 (1.02–1.1)	<0.001	1.1 (1.0–1.1)	<0.001	1.1 (1.03–1.1)
Plasma fibrinogen levels^f^	0.002^b^	1.4 (1.1–1.8)	0.04	1.3 (1.01–1.6)	0.001	1.4 (1.1–1.8)	0.01	1.4 (1.1–1.2)

Abbreviation: HR (95% CI)=hazard ratio (95% confidence interval).

a Kaplan–Meier analysis.

b Multivariate Cox regression model.

c FIGO I *vs* FIGO II *vs* FIGO III and IV.

d G1 *vs* G2 *vs* G3.

e Endometrioid *vs* non-endometrioid histology.

f Plasma fibrinogen levels per 100 units.
